# Age differences in spontaneous cerebrovascular reactivity at rest

**DOI:** 10.1002/alz70855_101198

**Published:** 2025-12-23

**Authors:** Allison C Engstrom, Arunima Kapoor, Shubir Dutt, John Paul M Alitin, Trevor Lohman, Aimée Gaubert, Isabel Sible, Anisa J Marshall, Fatemah Shenasa, Jillian L. Joyce, Xingfeng Shao, Danny JJ Wang, Daniel A. Nation

**Affiliations:** ^1^ University of California, Irvine, Irvine, CA, USA; ^2^ Memory and Aging Center, UCSF Weill Institute for Neurosciences, University of California, San Francisco, San Francisco, CA, USA; ^3^ Leonard Davis School of Gerontology, University of Southern California, Los Angeles, CA, USA; ^4^ University of Southern California, Los Angeles, CA, USA; ^5^ Mark and Mary Stevens Neuroimaging and Informatics Institute, University of Southern California, Los Angeles, CA, USA; ^6^ Keck School of Medicine, University of Southern California, Los Angeles, CA, USA

## Abstract

**Background:**

Cerebrovascular reactivity (CVR), a marker of cerebrovascular function, is important in regulating blood flow in response to vasoactive stimuli. Deficits in spontaneous CVR may indicate vulnerability to vascular dysfunction and cognitive impairment. As the population continues to age, understanding age‐related changes in cerebrovascular health is critical. Prior findings on age‐group differences in CVR are mixed, and no studies to date have investigated fluctuations in spontaneous CVR at rest.

**Method:**

A sample of independently living older adults (*n* =  129, mean age = 70.0; *SD* = 7.7; age range 55‐90 years, 68.2% female) and younger adults (*n* =  24, mean age = 22.5; *SD* = 2.4; age range = 18‐30, 83.3% female) free of dementia, clinical stroke, and major neurological or psychiatric disorder were recruited from the community and underwent brain MRI and clinical interview. Pseudo‐continuous arterial spin labeling MRI and capnography was used to quantify cerebral perfusion while at rest. Cerebral perfusion and etCO2 values were used to quantify whole brain and regional spontaneous CVR values which were compared in younger and older adult samples using ANCOVA and voxel‐based morphometry (VBM).

**Results:**

ANCOVA models revealed significantly diminished whole brain spontaneous CVR in older adults compared to younger adults (older adults mean = 21.06, *SD* =, 11.08, younger adults mean = 45.83, *SD* = 33.61, *F* = 44.76, *p* = 0.000000000057). Additionally, older adults had significantly lower spontaneous CVR in all regions, except the thalamus (*p* = 0.06), with regions in the medial temporal lobe having the largest effect sizes. At the voxel level, VBM reveled regional clusters in which older adults exhibited significantly lower CVR responses compared to younger adults.

**Conclusion:**

Independently living and cognitively healthy older adults showed reduced whole brain and regional spontaneous CVR compared to younger adults. The observed age‐group differences in spontaneous CVR at rest suggests that changes in cerebrovascular function may occur earlier in the lifespan and predate irreversible neuronal damage and cognitive decline. Further research is needed to determine if spontaneous CVR may be used as a valuable biomarker for age‐related cognitive decline, and to determine the age range at which spontaneous CVR responses begin to decline.

